# Transcatheter occlusion of multiple pulmonary arterio venous malformations

**DOI:** 10.4103/0974-2069.74062

**Published:** 2010

**Authors:** Prabhu C Halkati, Suresh V Patted, MD Dixit, Ravikant Patil

**Affiliations:** Department of Cardiology, KLE University, Belgaum, Karnataka, India

**Keywords:** Multiple, pulmonary arterio venous malformations, transcatheter

## Abstract

Pulmonary arterio venous malformations are one of the rare vascular anomalies. Usually single large pulmonary arteriovenous fistula (PAVF) can be closed through transcatheter route but multiple PAVF require surgical lobectomy. We describe a case of an 8-year-old boy with multiple PAVF, who underwent successful transcatheter closure with multiple coils.

## INTRODUCTION

Pulmonary arterio venous malformations is one of the rare vascular anomalies comprising direct communication between the branches of pulmonary artery and pulmonary vein, without intervening pulmonary capillary bed.[1] It was first described by Churton in 1897. Clinical presentation ranges from incidental finding on chest X-ray to cyanosis, heart failure, or neurological deficit. High incidence of neurological complications in the form of cerebral embolism or abscess mandates aggressive therapy whenever possible.

## CASE REPORT

**Figure 1:**
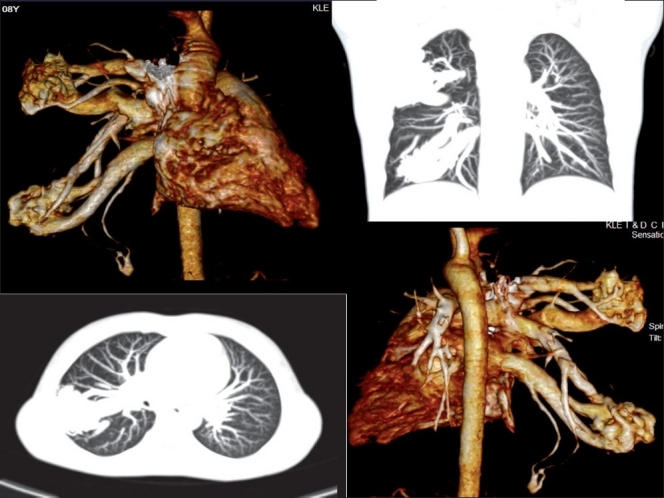
CT pulmonary angiogram showing multiple pulmonary arterio venous malformations

An 8-year-old boy presented with NYHA class II breathlessness since 5 years. He was evaluated at the other institute and all intra cardiac anomalies were ruled out. Patient had central cyanosis and grade III clubbing. There were no telangectasia of skin or mucous membrane. Physical examination was unremarkable with normal heart sounds and no murmurs. His oxygen saturation, as recorded on the pulse oxymeter, was 87% while breathing room air. Hemogram revealed hemoglobin of 19.3 mg/dl and packed cell volume (PCV) of 68%. His electrocardiogram was normal. Contrast echocardiogram showed appearance of contrast in the in the left atrium after three to four cycles following the opacification of the right-sided chambers, suggesting the presence of pulmonary arteriovenous fistula (PAVF). Computed tomographic pulmonary angiogram revealed multiple PAVF in the right lung [Figure 1].

**Figure 2:**
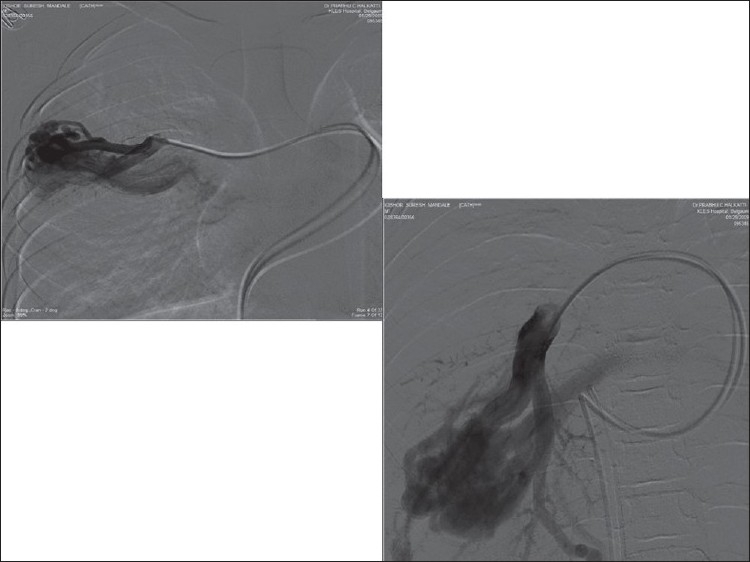
Conventional selective pulmonary angiogram

**Figure 3:**
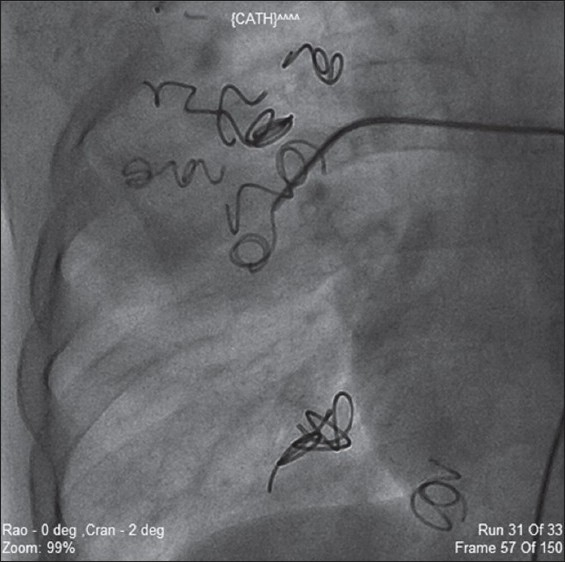
Deploying coils in pulmonary arterio venous malformations

The right femoral artery and vein were cannulated using 6F and 7F sheaths, respectively. Pulmonary angiogram, done using a 7F balloon tipped angiographic catheter, revealed a large PAVF in right lower lobe and another three smaller sized PAVF in upper lobe of right lung [Figure 2]. Using 6F internal mammary artery catheter, right lower lobe pulmonary artery was negotiated and a guide wire (0.035 × 260 cm, Terumo, Japan) was passed across the fistula. Then, 90-cm long 7F coil delivery system was positioned on the same wire. Check angiogram revealed the largest diameter of fistula to be 12 mm. A 15 mm × 8 cm coil (0.052", Cook Inc., Bloomington, IN, USA), which was 3 mm larger than the fistula diameter, was selected. With the help of introducer and delivery system, the coil was deployed into the fistula in a stable position. Since this did not occlude pulmonary AV fistula completely, another 8 mm × 8 cm coil was deployed close to the initial coil [Figure 3]. The check angiogram revealed tiny residual flow.

**Figure 4:**
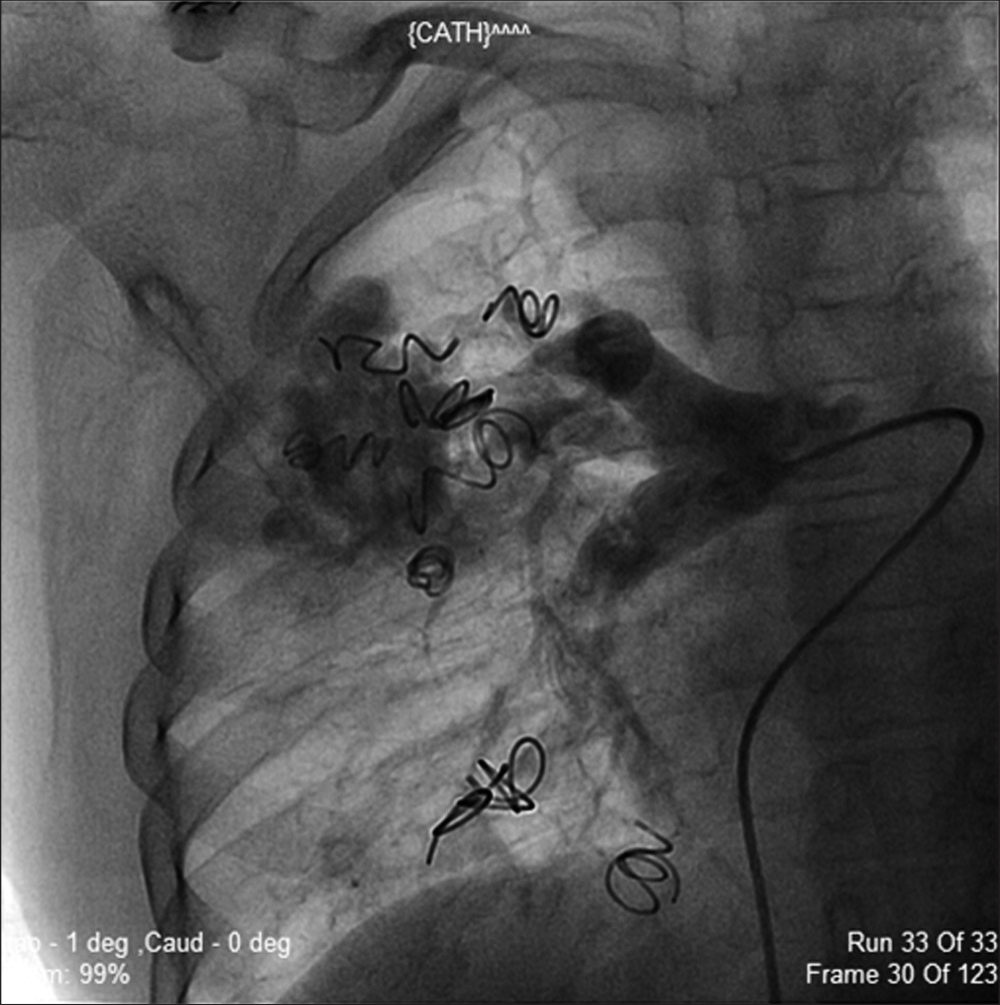
Post coiling of pulmonary arterio venous malformations

The other fistulae, one in the right lower lobe and three in the right upper lobe, were subsequently occluded in a similar manner using 8 mm × 8 cm, 5 mm × 5 cm, 5 mm × 6 cm, 8 mm × 5 cm (Cook Inc.) coils, respectively. The check angiogram revealed near complete occlusion of all the fistulae. The patient remained stable post procedure with an oxygen saturation of 97% while breathing room air, and was discharged. At 6-month follow up, a repeat pulmonary angiogram was done. It showed partial recanalization of the upper lobe fistula while the lower lobe fistulae continued to show complete occlusion. Room air oxygen saturation was 87%, but the patient continued to remain symptom free [Figure 4].

## DISCUSSION

PAVF, which occur more frequently in women, are transmitted as a dominantly inherited gene with incomplete penetrance. [3] They present with epistaxis, dyspnea or hemoptysis. Other presentations include headache in 43%, transient ischemic attack in 57%, cerebrovascular accidents in 18% and intra cerebral abscess in 33% of the cases.[1]

The morbidity associated with pulmonary arterio venous malformations is up to 50% in untreated patients as compared to 3% in those who receive treatment.[4] The goals of treatment are threefold: 1) improvement of dyspnea/hypoxemia 2) prevention of lung hemorrhage and 3) prevention of neurological sequel.

Transcatheter embolization is the preferred method of treatment of PAVF and can be done using stainless steel coils or detachable balloons. [5] Recently, vascular plugs have been used to occlude these abnormal communications. Coil embolization is particularly helpful for small or moderate-sized PAVMs while the large ones can be closed using a vascular plug or a duct occluder. Systemic embolization of the coils is one of the serious complications usually encountered when the PAVF has a torrential flow with no narrow point along its course. Using undersized coils can also increase the incidence of embolization. The rate of recanalization in PAVMs embolized with steel coils ranges from 10 to 57%. Most of the studies have shown that half of the recanalized PAVF were fed by bronchial artery branches. Thus, coil embolization should be performed as close as possible to the PAVF to avoid future development of bronchial artery-to-pulmonary artery anastomoses that may cause recanalization.[6] Occlusion of a PAVF with an Amplatzer vascular plug is a relatively newer and superior method. However, these devices are costly and using such devices for multiple PAVF, like in our case would increase the cost of the procedure several fold.[7]

Between 1942 and 1977, surgery was the only method of treatment for PAVF. Ligation, local excision, segmentectomy, lobectomy or pneumonectomy was performed in most cases. For multiple pulmonary AV fistulae, lobectomy was the method of choice until recently.[2] However, lobectomy has long-term problems in the form of development of pulmonary hypertension. In a 15-year prospective study, extensive lobectomy was shown to result in the early development of pulmonary hypertension in as high as 30% patients. As our patient was a young child, we decided to avoid extensive right lung lobectomy to prevent the development of pulmonary hypertension in future.[8] Recent advances in hardware have made transcatheter closure of these multiple PAVF feasible. Our case is an unique example of closure of multiple PAVF by transcatheter route. Though the procedure is time consuming and cumbersome, it has multiple advantages since there is no need for general anesthesia or thoracotomy. There is less pain, early recovery and absence of a scar. Also, there is no loss of normal lung tissue, which is likely to happen with a lobectomy or pneumonectomy.
